# Role of absolute versus relative voice rest in post-operative management of benign vocal fold lesions

**DOI:** 10.1017/S0022215124000835

**Published:** 2024-10

**Authors:** Mihika Sinha, Suresh Pillai, Shama Shetty, Usha Devadas

**Affiliations:** 1 Department of ENT, Kasturba Medical College, Manipal Academy of Higher Education, Manipal, India; 2 Department of Speech and Hearing, Manipal College of Health Professions, Manipal Academy of Higher Education, Manipal, India

**Keywords:** larynx, voice, dysphonia, informed consent, speech therapy

## Abstract

**Objective:**

Most otolaryngologists advocate absolute voice rest after laryngeal surgery, which proves difficult for patients, so we decided to evaluate the role of absolute voice rest versus relative voice rest in the post-operative management of benign lesions.

**Methods:**

Forty patients were recruited and divided in two groups: absolute voice rest and relative voice rest. Pre- and post-operative voice analysis (fundamental frequency, jitter, shimmer, voice handicap index, voice-related quality-of-life scale scores and compliance) were noted at one week and one month.

**Results:**

Voice analysis parameters including jitter (*p* = 0.035), shimmer (*p* = 0.020), voice handicap index (*p* < 0.001) and compliance (*p* < 0.001) were better in the relative voice-rest group. Frequency, number of voice breaks and voice-related quality of life showed no statistically significant results.

**Conclusion:**

There was no significant benefit of absolute voice rest on post-operative outcomes as determined by acoustic variables. Compliance and quality-of-life scores were low in the strict voice-rest group. Therefore, we should reconsider post-surgical voice-rest protocol.

## Introduction

Vocal fold pathologies are very common in otorhinolaryngology practice. Vocal folds are sensitive anatomical sites for injury, especially in patients with a history of voice abuse, overuse and misuse. Benign lesions are non-malignant growths of abnormal tissue on the vocal folds.

The common benign lesions of vocal folds are singer's nodule, polyps, papilloma, polypoidal degeneration (Reinke's oedema) and cysts^1^. Vibration of vocal folds due to regulated air passing through the larynx allows humans the ability to phonate. This is regulated through a complex neuromuscular, membranous and cartilaginous framework. Vocal-fold abnormalities, including masses, hamper the normal vibration. Scarring can occur after injury, inflammation or surgical interventions^2^. Vocal-fold scarring causes disruption of the well-structured lamina propria and patients present with significant hoarseness of voice^3^. Therefore, it is of paramount importance to avoid scarring due to trauma and inflammation, voice rest being one of the important recommendations to avoid this.

Voice rest during post-operative healing is advocated by otolaryngologists all over the world. However, a review of literature shows a lack of uniformity in the advice given to patients regarding voice rest and very few studies have been done to establish a fixed protocol. There is a challenge in making the patient comply with duration and type of voice rest, especially after an established diagnosis of benign histopathology. Through this study, we address this controversy, compare our findings with the studies worldwide and evaluate the role of voice rest in post-operative management of benign lesions of the vocal folds.

## Materials and methods

### Study design and setting

This is an observational cohort study. The study was carried out in the Department of ENT in a tertiary care centre in South India in the span of two years between the year 2020 and 2022.

### Study population and ethical approval

Forty patients with benign vocal fold lesions were recruited. Patients with a history of change of voice, confirmed to have a vocal fold lesion by rigid video-laryngoscopy, not benefitting from standard voice rest were included in the study. Patients with benign vocal fold lesions undergoing microlaryngeal surgery were included in the study. Patients matching the inclusion criteria underwent surgical intervention in the form of excision under general anaesthesia by microlaryngoscopy.

Patients excluded from the study were those with a history of previous surgery on vocal folds, patients with history of previous head and neck radiation and patients whose post-operative histopathology report came as malignant. Patients lost to follow up within the follow-up duration also were excluded. The study was approved by the institute's ethical committee prior to commencing (IEC number 77) and was registered with the clinical trials registry of India (REF/2021/06/034257).

### Data collection and statistical analysis

The patients were subjected to pre-operative analysis in the department of speech and hearing by a speech pathologist and pre-operative voice analysis in the form of measurement of fundamental frequency, shimmer, jitter and number of voice breaks. Patients also were requested to fill out voice handicap indices and voice-related quality-of-life (QoL) scales to assess the perception of their voice and its effect on their QoL. The patients were categorised into the two groups of absolute voice rest and relative voice rest, based on the choice of the treating consultant. Forty patients were followed up for one-month analysis with 20 in the relative voice-rest group and 20 in the absolute voice-rest group.

**Figure 1.**
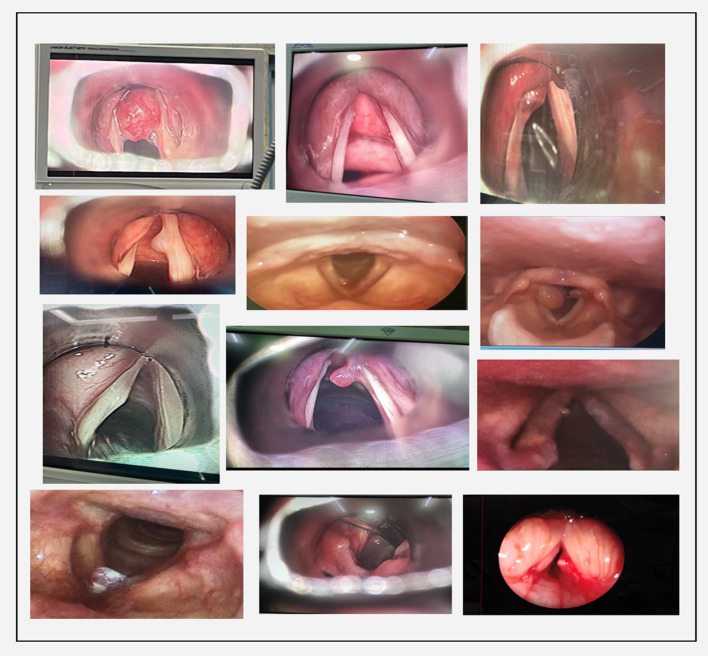
Intra-operative findings seen during microlaryngoscopy.

**Figure 2.**
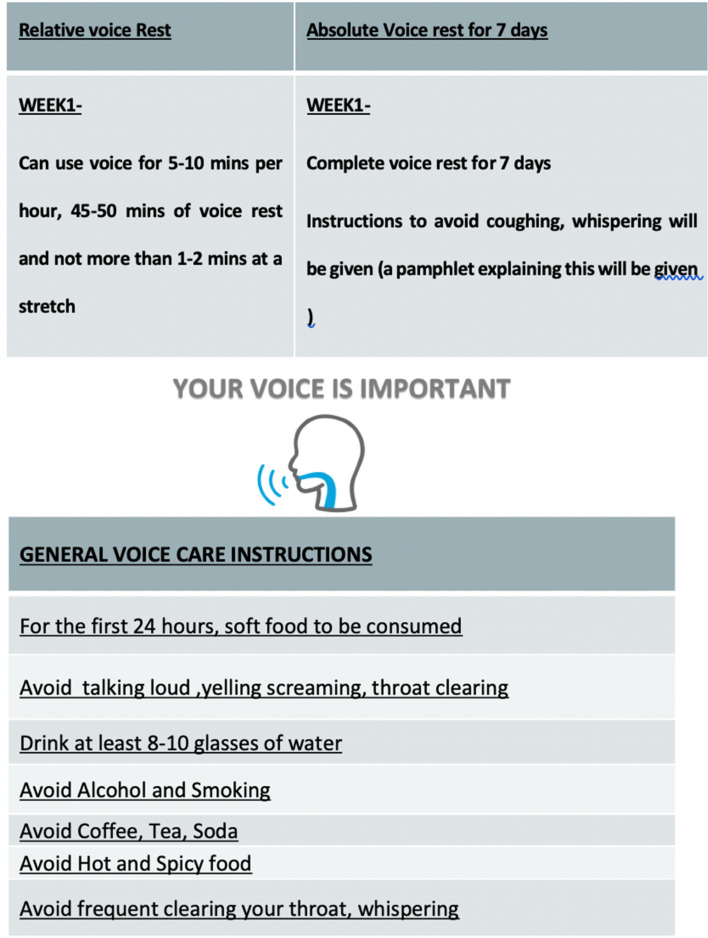
Pamphlet with instructions given to patients in relative and absolute voice-rest groups along with general voice hygiene instructions.

Patients underwent microlaryngoscopic surgery with cold steel instruments after confirming the intra-operative findings (Figure 1), and specimens were sent for histopathology studies. The patients were discharged one day after surgery on proton pump inhibitors (pantoprazole) and with a pamphlet (made by the authors as shown in Figure 2) mentioning the prescription of voice rest and instructions on general voice hygiene. The consultant of the treating unit decided whether absolute or relative voice rest was given (unit I consultants prescribed absolute voice rest and unit II consultant prescribed relative voice rest).

The patients were post-operatively assessed at one-week and one-month intervals with post-operative voice analysis along with voice handicap index, voice-related QoL and compliance to the prescribed voice rest on a five-point Likert scale. Primary outcome measurement was in the form of voice-analysis parameters (jitter, shimmer, fundamental frequency, number of voice breaks). Secondary outcome measures included voice handicap index, voice-related QoL scale, voice-rest instruction proforma and compliance using a five-point Likert scale.

Statistical analysis was performed using SPSS version 22 (IBM, Armonk, NY, USA) in order to detect the difference between the two groups. Repeated measures of analysis of variance (ANOVA) using PASS software (NCSS Statistical Software, Kaysville, UT, USA) was used keeping 90 per cent power, 5 per cent levels of significance and a standard deviation of 31.

## Results

### Primary Outcomes

#### Jitter and shimmer

While there was a significant difference in one-week values after surgery between the absolute and relative voice-rest groups (*p* = 0.035), no such significant difference was shown in the one-month values following surgery between the groups (*p* = 0.512). There was a significant difference in one-month post-surgical values between the groups (*p* = 0.020), but no such significant difference was shown in one-week post-surgical values between the groups (*p* = 0.289).

#### Number of voice breaks and fundamental frequency

Mann–Whitney U tests showed no significant differences in one-week post-op and one-month post-op between the absolute and relative voice-rest groups in number of voice breaks as *p* values were 0.841 and 0.565, respectively. Results revealed that there were no statistically significant differences between the groups as *p* values were greater than 0.05.

### Secondary Outcomes

#### Voice handicap index and voice-related quality of life (QoL) scale

**Figure 3.**
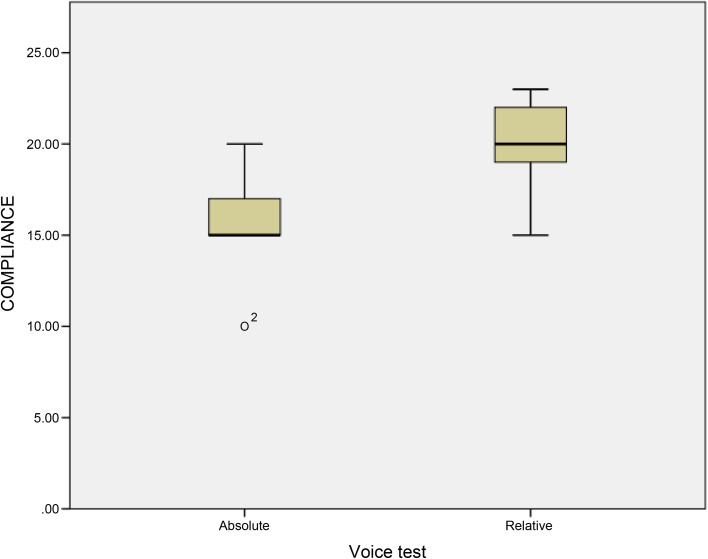
Box and whisker plot showing compliance in absolute and relative voice-rest group.

Voice handicap index, voice-related QoL and compliance were the secondary outcomes of this study. Results revealed that there were statistically significant differences between the groups as *p* values were 0.005 and less than 0.001 and the improvement in scores were found to be better in relative voice-rest group. Mann–Whitney U tests revealed no statistically significant differences between the groups as *p* values were 0.547 and 0.314 in one-week post-op and one-month post-op values, respectively. The box and whisker plot in Figure 3 depicts the distribution of compliance between the absolute and relative groups of voice rest. Compliance was found to be more in the relative voice-rest group than in the absolute voice-rest group.

## Discussion

### Definition of voice rest

There is no internationally accepted definition of what is meant by ‘relative voice rest’ nor is there a standardised protocol for the parameters of relative voice rest. Most of these definitions and parameters depend on the choice of the operating surgeon or are based on institution preference. In this study, the term ‘relative voice rest’ was defined as being “along with the generalised voice hygiene instructions mentioned in the pamphlet given to each patient, the patient can use the voice for 5–10 minutes per hour with 45–50 minutes of voice rest and not more than 1–2 minutes at a stretch.”

Because there is no standardised definition of what is meant by relative voice rest, it has been described differently by different authors. Whitling *et al*.^4^ allowed the relative voice-rest group to use voice for 7 days post-operative in a gentle, comfortable way and to avoid whispering and shouting. Kaneko *et al*.^2^ described relative voice rest as 3 days voice-rest period and compared the results to a complete 7 days voice-rest group and Kiagiadaki *et al*.^5^ described relative voice rest as 5 days of voice rest.

Another survey done by Coombs *et al*.^6^ revealed that ‘complete voice rest’ meant no voice production as per 86.5 per cent of respondents, while there was no consistent response as to how respondents described ‘relative voice rest’, and eight physicians were not aware of the term or had not put it to use in their clinical practice. There was a general agreement that a ‘relative voice-rest’ group should be given general instructions of no shouting, no singing, or whispering; however, several respondents also mentioned that they had their own ‘relative voice-rest’ regimes.

- Benign vocal cord lesions are common in ENT practice, but a standardised mode of post-operative voice-rest schedule has not yet been devised
- When one week of absolute voice rest instead of relative voice rest was advised after surgery, there was no discernible improvement in the quality of the voice as determined by acoustic variables and auditory analysis
- Poor adherence to lengthy and stringent voice-rest recommendations was observed
- Speech management needs to be revaluated and a relative voice-rest recommendation might increase compliance and produce better outcomes

### Comparison of voice parameters between the two groups

Our study evaluated jitter changes from pre-op values to one-week post-surgical and one-month post-surgical periods in both of the groups (absolute and relative). There was a significant difference in one-week post-op period values in case and control subjects (*p* = 0.035), but there was no such significant difference in one-month post-op values between the case and control subjects (*p* = 0.512).

Raju *et al*.^7^ conducted a prospective randomised control trial which was single-blinded involving 35 patients and categorised the patients into five- and two-day voice-rest groups. They found no statistically significant difference between the two groups, with the exception of jitter, where the five-day voice-rest group showed a statistically significant improvement over the two-day voice-rest group and found compliance was 43 per cent in the absolute voice-rest group of patients.^7^

A systematic review and meta-analysis done by Chi *et al*.^8^ compared four randomised control trials of 112 patients. Chi *et al*.^8^ reported comparable voice handicap index and acoustic variables in the form of jitter, shimmer and maximum phonation time in shorter- and longer-term voice-rest groups and unfavourable outcomes on QoL and compliance in the longer-term voice-rest group. In contrast, Cohen *et al*.^9^ reported results on 167 patients (a cohort study done both prospectively and retrospectively in a combined way and approximately equally divided into two groups of standard and no voice-rest groups). They noted that voice handicap index scores and acoustic variables showed no difference between the voice-rest and no-voice-rest groups in shimmer (*p* = 0.9590), jitter (*p* = 0.5692) or harmonic-to-noise ratio (*p* = 0.1871) which was statistically significant, and concluded that quality of voice and healing of wound post-operatively were similar in both groups and that ‘no voice rest’ gave equally good results.^9^

A prospective study of 55 patients by Singh *et al*.^10^ concluded that histopathologically the most common lesions were vocal fold cyst (20) vocal fold polyps (17), papilloma (6) and vocal nodules (7) in 40 per cent of the patients 30–40 years of age and 34 per cent 40–50 years of age and. We found most of our study population to be 40–50 years of age and vocal fold polyps (28) to be the most common histopathology diagnosis. The mean voice handicap index score pre-surgically was 88.15, which reduced to 26.5 after 3 months post-surgically, showing a statistically significant (< 0.001) improvement.^10^ This is similar to our study in which statistically significant differences were found between the voice handicap index scores of various sessions in absolute and relative groups separately (*p* < 0.001).

Dhaliwal *et al*.^11^ conducted a randomised controlled trial with 30 patients (15 in each group) and found that post-operative voice handicap index scores and secondary outcomes were not significantly different in the two groups. They ultimately argued that there is no advantage of voice rest on post-operative voice measurements and parameters as determined by patient self-perception, acoustic variables, and auditory-perceptual analysis. In contrast, we found statistically significant differences in the absolute voice-rest and relative voice-rest groups when it came to certain primary outcomes (shimmer, jitter) and secondary outcomes (voice handicap index and compliance), which makes relative voice rest a preferred prescription.

Owing to the fact that some amount of mechanical stimulation in the early stages helps in functional recovery of the vocal folds, 31 patients were recruited and were divided into two groups of three-day and seven-day voice rest in a randomised controlled trial done by Kaneko *et al*.^2^ They found that voice-analysis parameters (jitter, shimmer, and voice handicap index) were significantly better in the three-day group at one month post-surgical intervention. The data suggest that subjects who were in the relative voice-rest category (three days of voice rest followed by voice therapy) did better in terms of wound healing of the vocal fold and had generally better post-operative outcomes as compared to patients put on seven days of absolute voice-rest therapy.

Out of the 43 patients analysed in the retrospective study done by King *et al*.,^12^ 13 patients were put in the seven-day absolute voice-rest group, 15 were put in the less than seven-day voice-rest group, with voice handicap index scores noted during the pre-operative period once and twice in the post-operative phase. King *et al*.,^12^ found improvement in voice handicap index scores post-operatively amongst all patients and voice handicap index outcome did not change with the difference in the voice-rest recommendation in the different groups. Our study had similar results in which voice handicap index improvement was observed in all cases post-operatively in both the groups. In addition, significant differences in voice handicap index scores were noted from pre-op to one-week post-surgical and one-month post-surgical values in absolute voice-rest and relative voice-rest groups, with statistically significant difference between the two groups (*p* values 0.005 and < 0.001, respectively).

## Compliance

**Figure 4.**
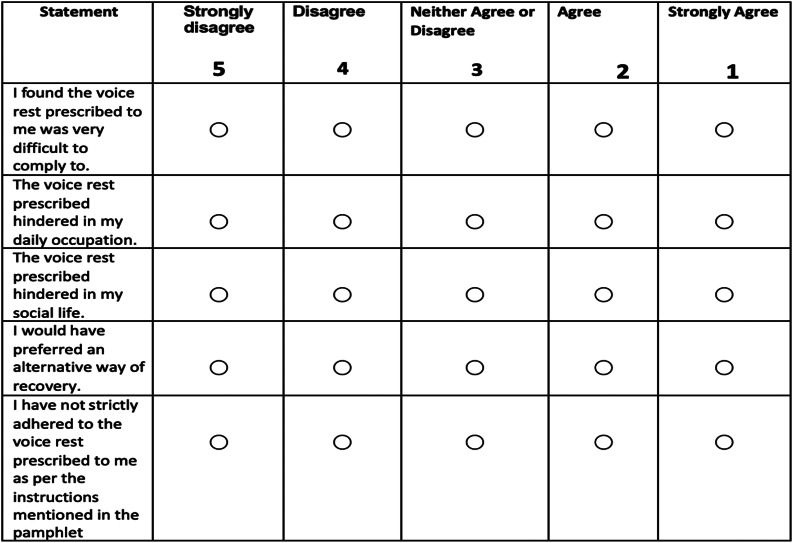
Five-point Likert scale developed by the authors to study the compliance of the patients.

A five-point Likert scale was used to study compliance of the patients in the two groups. The Likert scale, which was developed by the authors (Figure 4) as per the input of the ENT surgeons and speech and hearing pathologists, included factors such as whether the voice rest hindered their occupation, social life, and if they would have preferred an alternate way of prescription. The patients filled-out the scale survey at the first follow up post-surgically.

Based on our study, as noted at the end of one week on the Likert scale, there were statistically significant differences found in compliance between the absolute and relative groups of voice test (*p* < 0.001). Rousseau *et al*.^13^ determined compliance in their study by having the patient answer “never” to whether “I used my voice while on voice rest” and found that only 34.5 per cent of patients were compliant with voice rest, 25.5 per cent of noncompliant patients used their voice sometimes and 5.5 per cent did not comply with the voice rest at all (self-reported compliance was found to be low).

Twenty patients that were divided into absolute voice-rest and relative voice-rest groups were analysed by Whitling *et al*.^4^ for their compliance in a preliminary randomised, prospective, blind clinical trial after surgery for benign vocal fold lesions. They found that patients in the absolute voice-rest group found it more difficult to comply with the voice-rest instructions than patients in the relative voice-rest group. Compliance was higher in the relative voice-rest group, as measured by five-point Likert scale, than in the absolute voice-rest group.

## Conclusion

The results of this prospective study, in which the quality of voice was assessed in relation to the duration and type of voice rest following microlaryngeal surgery for benign vocal fold lesions, suggest three major outcomes. (1) When one week of absolute voice rest instead of relative voice rest was advised after surgery, there was no discernible improvement in the quality of the voice as determined by acoustic variables and auditory analysis. (2) Poor adherence to lengthy and stringent voice-rest recommendations was observed. (3) Acoustic factors and auditory analysis were used to establish the results, which showed that the timing of post-operative rest and speech management needs to be revaluated and that a relative voice-rest recommendation might increase compliance and produce better outcomes.

Through this study we have re-evaluated the practice of absolute voice rest, with an emphasis on less-debilitating methods for post-operative voice recovery. Hence, we need to reconsider post-operative speech management and switch over to relative voice rest which yields better or similar results.
